# Bile Acid Sequestrants Based on Natural and Synthetic Gels

**DOI:** 10.3390/gels9060500

**Published:** 2023-06-19

**Authors:** Magdalena-Cristina Stanciu, Marieta Nichifor, Carmen-Alice Teacă

**Affiliations:** 1 Natural Polymers, Bioactive and Biocompatible Materials Department, “Petru Poni” Institute of Macromolecular Chemistry, 41A, Gr. Ghica-Voda Alley, 700487 Iasi, Romania; nichifor@icmpp.ro; 2 Center for Advanced Research in Bionanoconjugates and Biopolymers, “Petru Poni” Institute of Macromolecular Chemistry, 41A, Gr. Ghica-Voda Alley, 700487 Iasi, Romania; cateaca@icmpp.ro

**Keywords:** bile acid sequestrants, hypercholesterolemia, natural and synthetic gels, structure, properties

## Abstract

Bile acid sequestrants (BASs) are non-systemic therapeutic agents used for the management of hypercholesterolemia. They are generally safe and not associated with serious systemic adverse effects. Usually, BASs are cationic polymeric gels that have the ability to bind bile salts in the small intestine and eliminate them by excretion of the non-absorbable polymer–bile salt complex. This review gives a general presentation of bile acids and the characteristics and mechanisms of action of BASs. The chemical structures and methods of synthesis are shown for commercial BASs of first- (cholestyramine, colextran, and colestipol) and second-generation (colesevelam and colestilan) and potential BASs. The latter are based on either synthetic polymers such as poly((meth)acrylates/acrylamides), poly(alkylamines), poly(allylamines) and vinyl benzyl amino polymers or biopolymers, such as cellulose, dextran, pullulan, methylan, and poly(cyclodextrins). A separate section is dedicated to molecular imprinting polymers (MIPs) because of their great selectivity and affinity for the template molecules used in the imprinting technique. Focus is given to the understanding of the relationships between the chemical structure of these cross-linked polymers and their potential to bind bile salts. The synthetic pathways used in obtaining BASs and their in vitro and in vivo hypolipidemic activities are also introduced.

## 1. Introduction

Hydrogels are polymeric networks, derived from nature or synthesized, formed through cross-linking processes, and possess a specific attribute relying on capacity to retain large amounts of water and to maintain 3D hierarchical structures. These structures are controlled on a molecular level by gelation chemistry principles (chemical or physical interactions including physical entanglement, electrostatic interactions, metal coordination, or covalent cross-linking) which provide them well-designed properties for different applications [1,2,3].

Hydrogels can be classified [2] based on composition and derivation, as:

- biological hydrogels
- synthetic hydrogels: organic (neutral, ionic, and conductive polymers, based on the types of charged pendant groups to or within the main polymer chain) or inorganic
- hybrid hydrogels (combining the specific features of both natural and synthetic polymers from their formulations).

The natural polymers, namely, polysaccharides and proteins, are the most widely investigated for the preparation of hydrogels, mainly through chemical cross-linking methods such as Schiff base reaction, epoxide coupling, addition reaction, click reaction, condensation reaction, and free radical polymerization (redox/photopolymerization), as well as other methods, such as genipin coupling and polycarboxylic acid-based esterification cross-linking [4]. Monomer/polymer functional group and backbone chemistry, cross-linking agent chemistry and density, functional additives, and synthesis environment (e.g., temperature, pH, ionic strength) are among the main factors of influence for the preparation of hydrogels [5,6].

**Figure 1:**
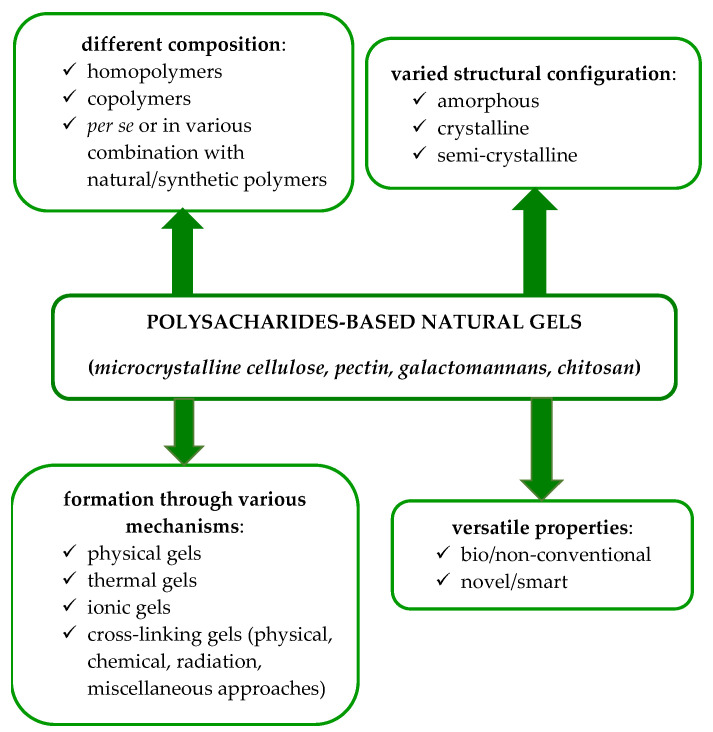
Information related to polysaccharide-based natural gels (adapted and redrawn from [7]).

In the last few years, an attractive approach based on combinations of different polysaccharides with each other or with other polymers (natural and/or synthetic) has emerged in the research area of polysaccharide-based gels. A schematic representation of some important issues related to these gels is given in Figure 1.

Natural polymers (e.g., vegetal oils; gelatin and silk fibroin (proteins of animal origin); vitamins; etc.), synthetic polymers, micro-/nanosized particles, and/or fibers are effectively employed in order to produce polysaccharide-based formulations and materials with enhanced or novel properties, which make them suitable for a more comprehensive range of applications [8,9,10,11,12,13]. A large range of strategies applied in order to design polysaccharide-based hydrogels with ordered structure were recently reviewed [14] with a focus on structural architectures with multistratified and intelligent features that are generally observed in materials from nature and provide broad potential in pharmaceutical and biomedical applications. Many exhaustive research studies have considered global-level applications of hydrogels, including biomedical, pharmaceutical and environmental. Some examples are presented in Table 1.

Atherosclerosis is the main cause of morbidity and mortality in developed countries [15]. One of the most effective and employed methods for clinical treatment and alleviation is implant intervention, i.e., stents. Surface modification has an excellent improvement effect for cardiovascular stents in terms of physicochemical properties and biocompatibility issues [16]. In this context, multifunctional coatings composed of hyaluronic acid (HA) and polydopamine (PDA) can be efficiently applied for a large range of cardiovascular biomaterials’ modification, particularly magnesium alloys, with a positive impact when interacting with the PDA/HA coatings and the surrounding microenvironment from the cell regulation and corrosion point of views.

**Table 1:** Some examples of possible applications for hydrogel formulations.

Possible Applications	Observations	Reference
BASs	drug carrier formulations that are based on electrostatically assisted assembly; biocompatible block copolymers and biosurfactants (such as bile salts) are particularly interesting, as well as surface-functionalized layered double-hydroxide nanocontainers as BASs for lowering hyperlipidemia; the most promising BASs are biopolymer-based BASs, mainly those based on polysaccharides	[14,17,18,19]
sustainable agriculture	hydrogels, and sensors for controlled agrochemical release, promoting growth, soil conditioning, sensing of soil condition, and other combined multiple actions in agriculture	[20]
energy storage devices (batteries, supercapacitors)	hydrogels should be paintable, elastic, transparent, super-safe, bio-friendly for customized structural energy storage (ultrathin and flexible, mobile appliance, weavable) and transparent attachable storage (artificial organs, digital health analyzers, wearable devices)	[21]
solar water purification	hydrogel-based evaporators (polymeric networks tailored to regulate the water state as a function of functional additives, monomer/polymer functional groups, cross-linking density); their micro- or nanostructure and surface topography can be also tailored by interfacial synthesis, freeze-drying, and surface coating	[22,23,24]
drug delivery	in situ forming delivery systems (e.g., bioorthogonal cross-linking strategies); multicomponent hydrogels; stimuli-responsive hydrogels; nanogels; release of therapeutics from 3D printed hydrogels	[25,26,27]
wound healing	advanced functions of hydrogel dressings such as: antimicrobial property, adhesion and hemostasis, anti-inflammatory and antioxidation, substance delivery, self-healing, stimulus response and the recently emerged wound monitoring feature; new wound healing devices can combine smart hydrogel dressings with physical therapies	[28,29,30]

Risk factors in cardiovascular diseases are high levels of total cholesterol (TC) and low-density lipoprotein (LDL). The latter (“bad” cholesterol) transports cholesterol from the liver to the tissues through the blood vessels, where, over time, it can form atheroma plaques, causing atherosclerosis. High-density lipoprotein (HDL) (“good” cholesterol) mediates the transport of cholesterol from cells in arterial tissues, particularly from atherosclerotic plaques, to the liver. TC is calculated by summing HDL, LDL and 20% of the triglyceride (TG) value. Clinical statin trials showed that reducing of LDL by 1% determines the decrease in risk of atherosclerotic cardiovascular disease. Therapeutic options for lowering TC and LDL are a healthy lifestyle and lipid-lowering drug therapy. Statins, BASs, niacin, fibrates or cholesterol absorption inhibitors (ezetimibe) are among the drugs used for the treatment of hyperlipidemia. This review focuses on recent BAS synthetic routes, results concerning their in vitro BA adsorption, and in vivo tests of cholesterol reduction.

## 2. Lipid-Lowering Drugs

Lipid-lowering agents, also sometimes termed hypolipidemic agents, cholesterol-lowering drugs, or antihyperlipidemic agents, are a varied group of pharmaceuticals that are used to lower the level of different forms of lipids in patients with hypercholesterolemia. The various classes of lipid-lowering drugs have different influences on the cholesterol profile and adverse effects. Clinically, the selection of the medication relies on the cholesterol profile, cardiovascular risk, and the liver and kidney functions of the patient, assessed versus the balance between risks and benefits of the drugs. As the level of lipids is crucial for cardiovascular diseases, the medication diminishes circulatory disorders. Types of cholesterol-lowering drugs include, among others, statins, niacin and BASs.

### 2.1. Statins

Statins are the drugs of choice in the treatment of hypercholesterolemia. They can directly block the synthesis of cholesterol in the liver by inhibiting 3-hydroxy-3-methylglutaryl coenzyme A (HMG-CoA) reductase activity [31,32,33,34,35,36,37]. This coenzyme slows the production of LDL and lowers its level in the body. Consequently, statins reduce the risk of serious cardiovascular incidents, with their high degree of mortality. Statins are not recommended for pregnant women, nursing mothers, or patients with hepatic dysfunctions. Among the adverse effects of statins are muscle damage (pains, cramps), hepatotoxicity, augmentation of blood sugar level, memory disorders, gastrointestinal symptoms (flatulence, constipation, nausea, diarrhea), and skin rashes.

### 2.2. Niacin (Nicotinic Acid, Vitamin B3)

Pyridine-3-carboxylic acid was the first drug used in the 1950s for a decrease of LDL level. Niacin, at a dosage of 3 g per day, increases HDL by 15–35%, decreases TG by 20–45% and LDL by 5–21% [38]. Vitamin B3 increases triglyceride-rich lipoprotein catabolism and decreases fatty acid flux to the liver, being an antagonist of hydroxycarboxylic acid receptor 2 (HCA2). Skin redness, nausea, stomach irritation, liver damage, gout, and blurred vision are among the side effects produced by niacin [39]. In certain clinical circumstances, such as mixed dyslipidemia resistant to statin therapy or intolerance to statins, niacin may be helpful [38].

### 2.3. Bile Acid Sequestrants

BASs were among the first drugs accepted for treatment of high blood cholesterol, but nowadays they are not among the more often used drugs for management of hypercholesterolemia. However, due to many side effects occurring during treatment with statins, plus BASs’ favorable activity against other diseases, for example, diabetes, the clinical use of BASs has begun to be reevaluated [40].

#### 2.3.1. Bile Acids

**Figure 2:**
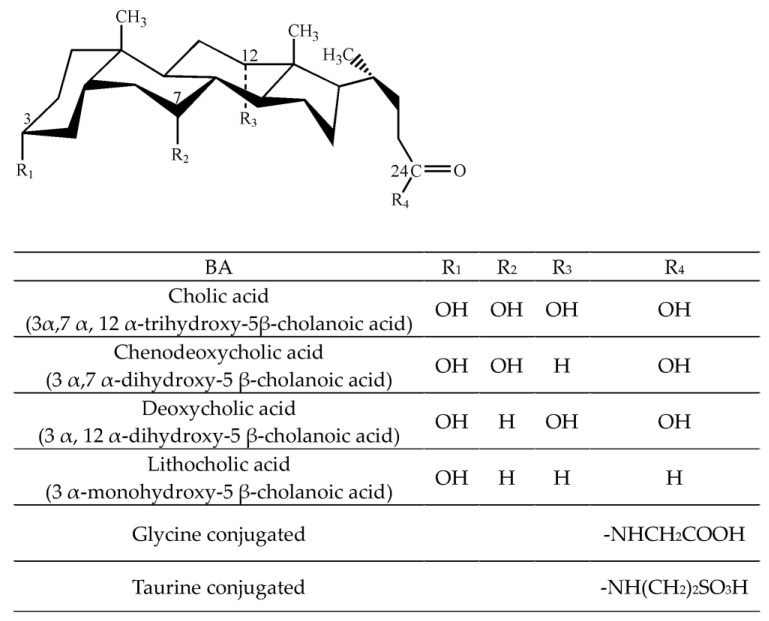
Primary and secondary BA.

These are natural compounds found in bile along with cholesterol, lecithin, bile pigments and water. They contain a rigid hydrophobic steroid nucleus formed by four condensed rings, marked A, B, C (with six C atoms) and D (with five C atoms), as well as a flexible side alkyl chain terminated with a carboxyl group. Cycles A and B are linked in *cis* configuration, while cycles B-C, C-D are bound in *trans* configuration (Figure 2) [41,42,43,44,45,46,47]. The binding in *cis* position of cycles A and B determines a curvature of the steroid skeleton, which allows the existence of two faces: α and β [48,49]. The α face is completely hydrophobic due to the presence of the three methyl groups, while the β face of the steroid cavity is hydrophilic due to OH groups converging towards the cavity with the end carboxylic group. Because of their facial amphiphilicity, BAs are able to form micelles or other supramolecular structures in a stepwise manner, due to hydrogen bonds and back-to-back hydrophobic interactions [50,51,52].

The synthesis of BA takes place in liver cells (hepatocytes). The resulting acids are primary (cholic and chenodeoxycholic acids) that have been synthesized from cholesterol conversion catalyzed by hepatic microsomal cholesterol 7α-hydroxylase. In the liver cells, the conjugation of primary BA with glycine (75%) and taurine (25%) takes place, leading to conjugated BA. The conjugated acids are stored in the gallbladder. When the digestion process begins, they are released from the gallbladder through the bile duct into the small intestine. Here, due to intestinal pH value (7–9), BAs are in the protonated form, which increases their solubility and fat emulsification capacity. Secondary BAs (deoxycholic acid and lithocholic acid) are obtained by 7α-dehydroxylation of the conjugated BA in the intestinal lumen followed by splitting of glycine and taurine groups. Cholic acid is transformed into deoxycholic acid, while chenodeoxycholic acid is converted into lithocholic acid. BAs are reabsorbed through the intestinal epithelium of the terminal ileum and reach the liver through the portal vein. This circuit of BAs in the body constitutes the enterohepatic circulation and has the role of maintaining a constant level of BAs in the body. Overall, 95% of the production of BAs ends up being reused through the enterohepatic circuit, the remaining 5% being lost through excretion. The loss of BAs is compensated by their de novo synthesis in the hepatocytes.

#### 2.3.2. Bile Acid Sequestrants: Characteristics and Mechanism of Action

BASs are cross-linked positively charged polymeric materials that control cholesterol levels in an indirect way by their ability to bind BA salts in the small intestine [53,54,55,56,57,58,59,60,61,62,63,64]. The BAS–BA complex is eliminated from the body through excretion, which causes the decrease in BA. In order to reestablish the BA level necessary for digestion, new amounts of cholesterol are modified in the liver, lowering the TC content in the bloodstream. BASs reduce the level of LDL by 15–30% and that of TG by 25%, increasing the level of HDL by 4–10% [33,65]. BASs are helpful in lowering the risk of cardiovascular events when used alone or in combination with other lipid-lowering medications (niacin, fibric acid derivatives, and statins) [35]. BASs must contain in their structure proper cationic charge density, long hydrophobic pendant groups, and adequate flexibility and capacity of swelling that favor BA adsorption, mainly through electrostatic and hydrophobic interactions. BASs can also reduce blood glucose in patients with diabetes due to deactivation of farnesoid X receptor in the presence of low levels of BA. This change causes the downregulation of enzymes related to hepatic insulin resistance and glucose intolerance and improves hepatic glucose utilization and glucose uptake [40]. Due to their oral administration and insolubility, which prevent permeability along the digestive tract, BASs are considered safe, without systemic adverse effects. Unfortunately, BASs already in clinical use can produce various side effects of gastrointestinal nature (flatulence, constipation, nausea, dyspepsia); therefore, the BAS treatment period is sometimes limited by patients’ poor tolerance.

#### 2.3.3. BASs in Clinical Use

**Figure 3:**
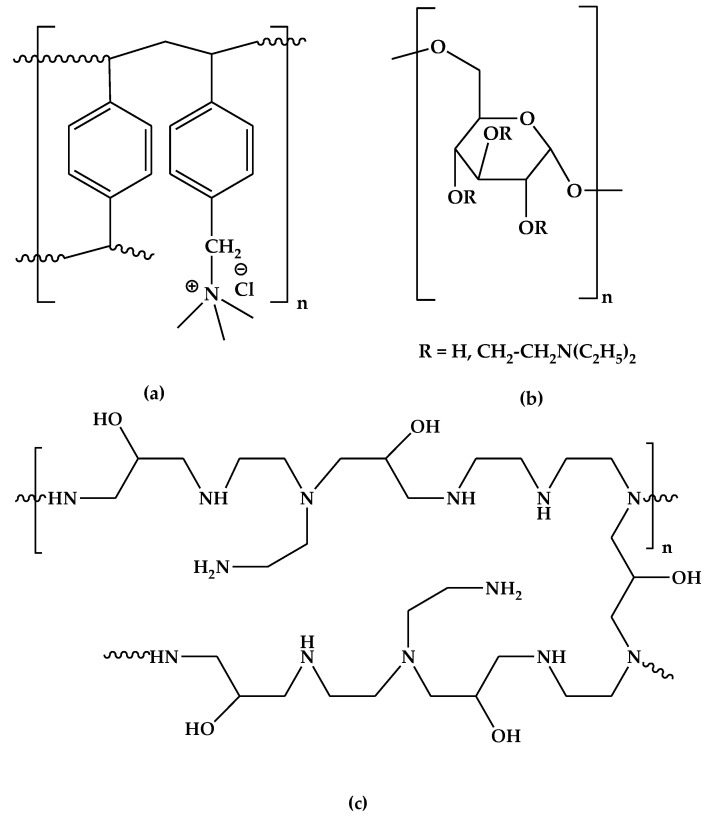
Chemical structures of cholestyramine (**a**), colextran (**b**) and colestipol (**c**).

Cholestyramine, colextran and colestipol are first-generation BASs [66]. Cholestyramine (Figure 3a) is a cationic hydrogel made of polystyrene partially cross-linked with divinylbenzene (2%) [67,68]. Cholestyramine is synthesized by chloromethylation of polystyrene cross-linked with divinylbenzene followed by quaternization with trimethylamine. Colextran (Figure 3b), an ether of dextran and diethylethanolamine, is an ion exchange resin that reduces both total cholesterol and triglycerides levels [69]. Colestipol (Figure 3c) is obtained by condensation reaction between diethylenetriamine and epichlorohydrin [70].

**Figure 4:**
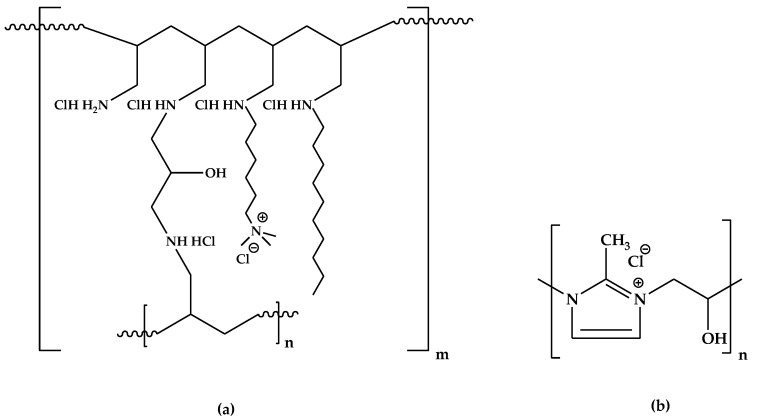
Chemical structures of colesevelam (**a**) and colestilan (**b**).

Because it contains secondary and tertiary amino groups in the chemical structure, its efficiency depends on the pH of the environment. Thus, at the pH of the small intestine (7–9), all amino groups are ionized. The above commercial resins preferentially bind dihydroxy BA from the gastrointestinal tract (GIT). Since the liver produces both dihydroxy and trihydroxy BA, the content of trihydroxy acids in the BA pool increases over time by using BASs, causing a decrease in BAS efficiency. Moreover, the stability of the BAS–BA complex is poor, leading to partial BA dissociation along the GIT, due to pH variation and presence of competitive anions. Consequently, high doses of first-generation BASs (16–24 g/day) are necessary to obtain a significant therapeutic response (20% cholesterol reduction), and hence the need to synthesize new compounds with increased binding capacity and affinity to bile salts. The drawbacks of first-generation BASs led to extensive research focused on the design of new more efficient cationic gels. From these gels, only colesevelam hydrochloride and colestilan (or cholestimide) (Figure 4) have been approved for marketing: colesevelam in the US under the trade name WelChol and colestilan in Japan with the trade name Cholebine [71,72,73,74,75,76]. These second-generation BASs have an increased affinity to bile salts and fewer adverse effects than first-generation BASs. Colesevelam (Figure 4a), having in its chemical structure both primary amino groups and quaternary ammonium salts, allows the binding of BA salts through electrostatic and hydrophobic interactions. Due to its chemical structure, colesevelam has affinity for both di- and trihydroxy BA, being three times more effective than cholestyramine and colestipol. Irritation of GIT and constipation are among the side effects of colesevelam. Colestilan (Figure 4b) contains imidazolium salts [77]. It is obtained by cross-linking of poly(2-methylimidazole) with epichlorohydrin. In vitro studies demonstrated for colestilan a superior capacity of binding BA salts compared to cholestyramine, due to its higher ion exchange capacity and water retention.

#### 2.3.4. BASs under Research

Many cationic hydrogels with various chemical structures and polymeric backbones have been reported and their performance as potential hypocholesterolemic agents tested at different levels, from in vitro BA binding until clinical trials. Synthetic polymers based on (meth)acrylates/acrylamides, alkylamines, allylamines and polysaccharides such as dextran, pullulan, methylan, chitosan, cellulose, poly(cyclodextrin) have been used to synthesize potential BASs. Unlike synthetic polymers, polysaccharides have been employed as a starting material to synthesize BASs due to their biocompatibility, low toxicity, hydrophilicity and the existence of hydroxyl groups that may be easily functionalized [78,79]. Another advantage of polysaccharides such as pectins, galactomannans, and chitosan is their intrinsic hypocholesterolemic activity due to both the influence on cholesterol bioaccessibility and bile salt binding capacity [80]. These properties can be significantly improved by amino group attachment. In the following, they are classified as a function of their polymeric backbone.

##### Poly(meth)acrylates and Poly(meth)acrylamides

**Figure 5:**
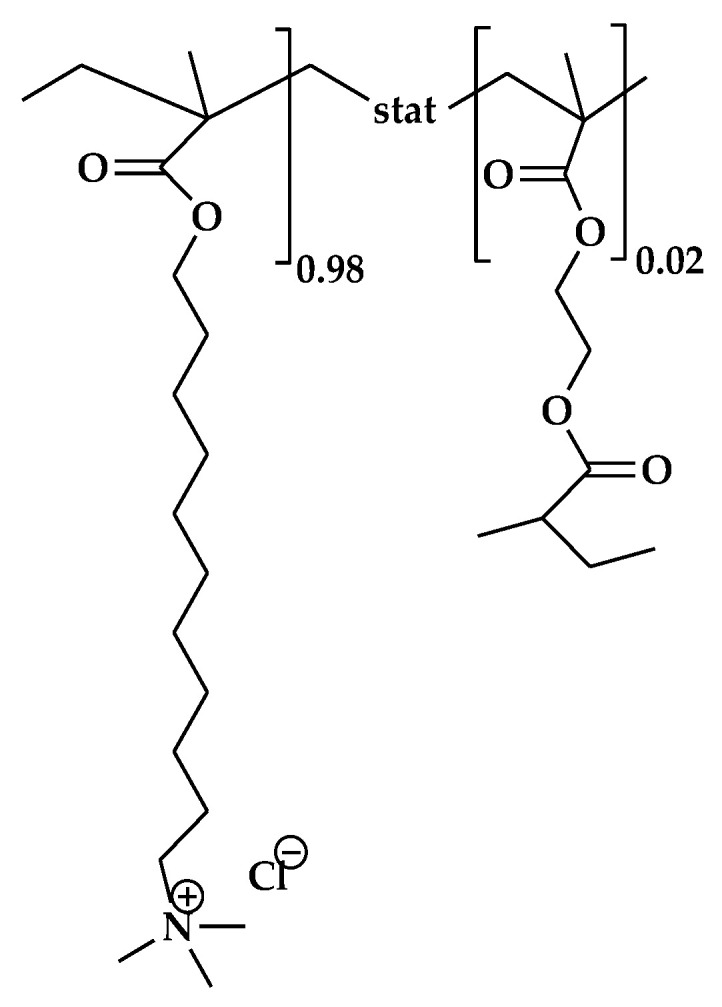
Chemical structure of SK&F 97426-A (adapted and redrawn from [81,82]).

SK&F 97426-A is the commercial term for the copolymer of 11-trimethylammonium decylmethacrylate chloride and ethylene glycol bismethacrylate (99:1) (*w*/*w*) (Figure 5) [81,82]. In vitro experiments showed that SK&F 97426-A and cholestyramine had similar binding capacities for tested BA (between 2.5 and 4 mmol/g) and bound a similar proportion of the dihydroxy BA. SK&F 97426-A had much higher affinity for trihydroxy BA (glycocholic and taurocholic acids) than cholestyramine. Glycocholic acid and taurocholic acid dissociate from SK&F 97426-A more slowly (27% and 25%, respectively) than cholestyramine (89 and 84%, respectively). SK&F 97426-A showed 2–3 times higher capacity of elimination of bile salts and 2.1–3.4-times and 2.3–3.2-times greater decrease in TC and LDL + VLDL, respectively, compared to cholestyramine [83]. TGs were also reduced by up to 31% in 1 week with both BASs.

Poly[(3-acrylamidopropyl) trimethylammonium chloride] (PAMPTMA)-based hydrogels, synthesized by supplemental activator and reducing agent atom transfer radical polymerization (SARA ATRP), were tested for in vitro adsorption of sodium cholate in simulated intestinal fluid [84]. Ethyl 2-chloropropionate was used as initiator, while two diacrylates with different hydrophilicities (1,4 butanediol diacrylate and tetra(ethylene glycol)diacrylate) were employed as cross-linkers. SARA ATRP afforded a perfectly controlled polymeric structure, while modification of polymer composition and its cationic part allowed a change in polymer-binding ability. Furthermore, gels prepared by SARA ATRP show a more homogeneous structure that the ones obtained by classical free radical polymerization (FRP), due to the quick initiation and reversible deactivation reactions [85,86,87]. The cationic hydrogels were efficient BASs, their binding properties being comparable to one of the most effective commercial BASs, colesevelam hydrochloride, regarding binding affinity and maximum binding capacity.

**Figure 6:**
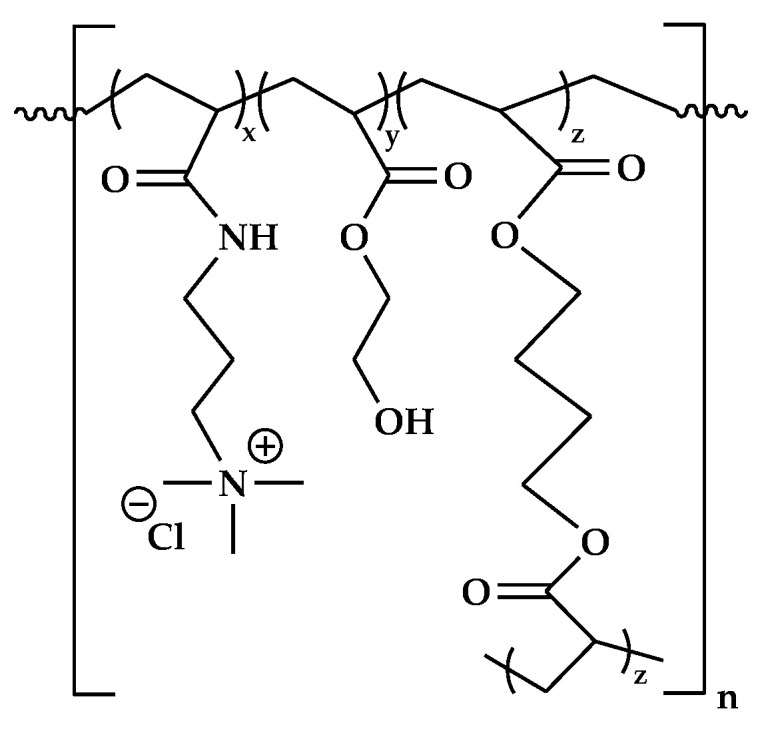
Chemical structure of hydrogels based on PAMPTMA and PHEA (adapted and redrawn from [88]).

New hydrogels based on PAMPTMA and poly(2-hydroxyethyl acrylate) (PHEA) (Figure 6) in various molar ratios were synthesized for an advanced study of the relationships between polymeric structure and BA adsorption properties [88].

The presence of hydroxyl groups in the gel (PHEA segment) afforded hydrogen bonds with bile salts. FRP and SARA ATRP were used to assess for the first time how the polymerization process affected the effectiveness of the BA adsorption. The findings suggested that electrostatic interactions were mostly responsible for the binding process because the performance of the gels decreased when PHEA content increased. The hydrogels formed by SARA ATRP had a much higher binding capacity than the hydrogel created by FRP, indicating that high-performance materials can be synthesized by advanced polymerization processes. Hydrogels created by FRP or SARA ATRP displayed a quick binding, equilibrium being reached in 30 min. SEC and ^1^H-NMR analysis showed that the hydrogel backbone did not degrade under the conditions used for in vitro degradation studies at 37 °C in simulated gastric fluid for 2 h (pH = 1.2) and simulated intestinal fluid for 3 h (pH = 6.8).

##### Polyalkylamines

**Figure 7:**
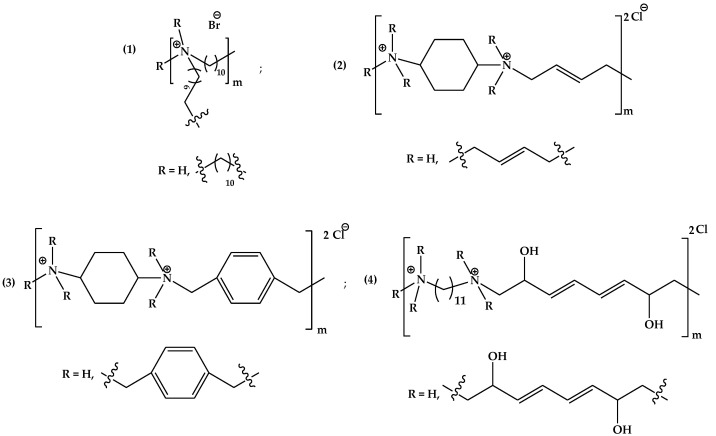
Polyalkylamine gels acquired by diamines and dihalo/diepoxy compounds (adapted and redrawn from [89]).

New hydrogels based on poly(alkylamines) were synthesized by the reaction between various diamines and dihalo compounds or diepoxides in DMF/methanol mixture in the presence of sodium carbonate, which improved the reaction times and yields (Figure 7) [89].

The N-substituted polymers obtained had a covalently cross-linked structure and displayed hydrogel behavior at low pH, due to the occurrence of polyammonium ions. At high pH, the polymers lost their swelling capacity because of the presence of the free base polyamine form. ^13^C-NMR, thermal analysis and swelling behavior revealed a highly branched structure that was slightly cross-linked. In vitro tests proved that cholic acid sequestrant ability was 10–15 times greater than that of cholestyramine. Furthermore, clinical investigations demonstrated the cholesterol-lowering effectiveness of polymeric materials in hypercholesterolemic patients.

**Figure 8:**
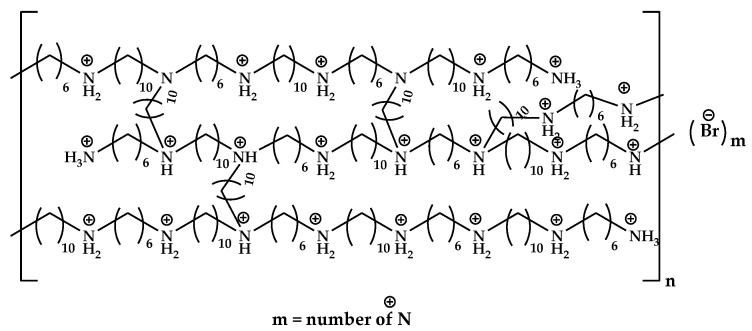
Chemical structure of DMP 504 gel (adapted and redrawn from [90]).

A novel BAS, DMP 504 (Figure 8), developed by the DuPont Pharmaceuticals Company, is a highly cross-linked polymer synthesized by the polymerization of 1,10-dibromodecane with hexamethylene diamine [90]. Cholate binding capacity of DMP 504 was determined via plotting the bound cholate mass versus the mass of DMP 504 after incubation of resin with a cholate solution of known concentration.

Reverse-phase HPLC was employed for analysis of the residual cholate solution. No changes in polymeric structure and no increase in extractable water-soluble polyamines (synthetic impurities and potential degradation products of the polymer) were identified in DMP 504 over 6 months’ storage at room temperature [91]. Equilibrium binding parameters for DMP 504 were established and compared to that of cholestyramine [92]. DMP 504 had double the binding capacity and a triple the affinity for glycocholate and glycochenodeoxycholate of cholestyramine. Comparable results were achieved for taurine conjugates of cholate and chenodeoxycholate. According to the mathematical model of human bile flow, DMP 504 would be around three times as effective as chitosan in a clinical trial. In vivo results of DMP 504 hypolipidemic activity were compared to those of cholestyramine [93]. DMP 504 increased sevenfold the excretion of BA and threefold the excretion of sterols in comparison with cholestyramine. DMP 504 decreased LDL by 15.8–34.1% after 2 weeks (for doses of 0.9–7.2 g/day) and by 0.8–19.7% after 6 weeks (for doses of 0.9–5.4 g/day), while cholestyramine diminished LDL by 28.3% after 2 weeks and by 23.9% after 6 weeks [94].

##### Vinyl Benzyl Amino Polymers

**Figure 9:**
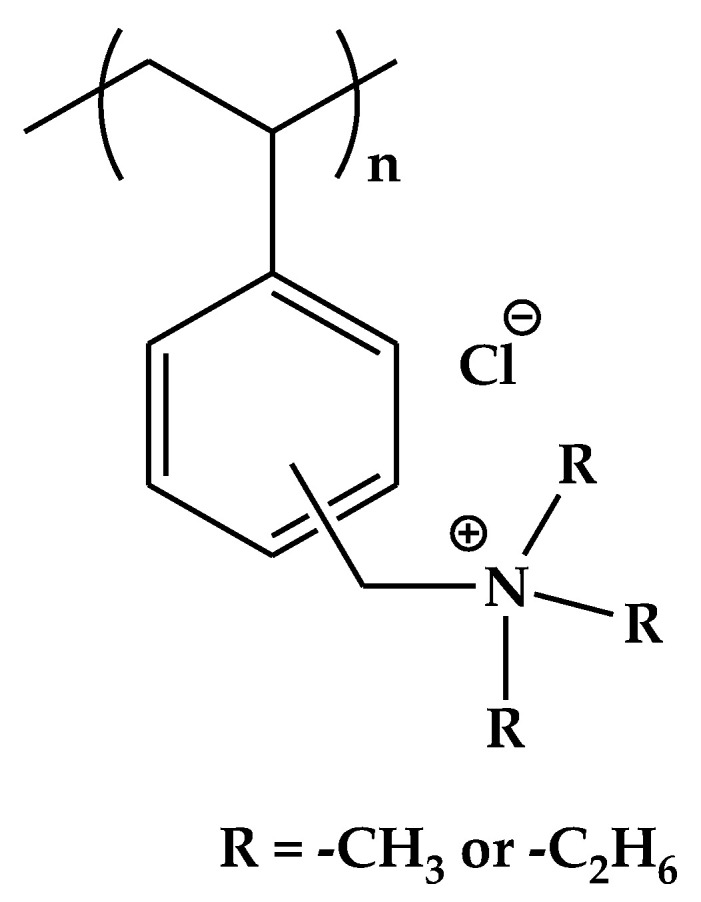
Polyalkylamines obtained by polymerization of vinylbenzyl-N,N,N-trialkylammonium chlorides (adapted and redrawn from [95]).

Starting from the classical structure of cholestyramine, based on vinyl benzene cross-linked with divinyl benzene, several attempts were made to improve this type of gel’s performance. Zarras reported that the polymerization in aqueous or organic solvents of monomers synthesized through the reaction of vinyl benzyl chloride with either tri-n-alkylamines (trimethylamine, triethylamine, tri-n-butylamine) (Figure 9) or salts based on 2-ionene oligomers afforded the formation of water-soluble polymers obtained in high yield and having high inherent viscosities [95].

The tested polymers had 18–27% better capacity than cholestyramine to bind BA. The results showed the best performance for the polymer based on trimethylamine (27% better than commercial BASs). The polymerization of the monomer resulted from reactions between divinylbenzyl and N,N,N,N-tetramethylethylenediamine, allowing the obtaining of a cross-linked product that showed the lowest (18% better than cholestyramine) ability to retain BA compared to water-soluble polymers.

Another interesting approach was reported by Zhang et al. [96] with the goal of improving BAS–BA affinity and slowing down BA release from the complex. For this purpose, a gel with the same chemical structure as cholestyramine was prepared, except that a certain amount (8–18%) of vinylbenzyl chloride groups was aminated with N^2^,N^2^-dimethylamino-N^1^-2-ethyl-choleamide instead of trimethylamine. Increased content in cholic acid moieties enhanced taurocholate ion binding and decreased its rate of desorption in the presence of 50 mM aqueous NaCl. This favorable effects were assigned to cooperative binding due to van der Waals/hydrogen bond interactions between covalently bound BA and ligand.

##### Polyallylamines

**Figure 10:**
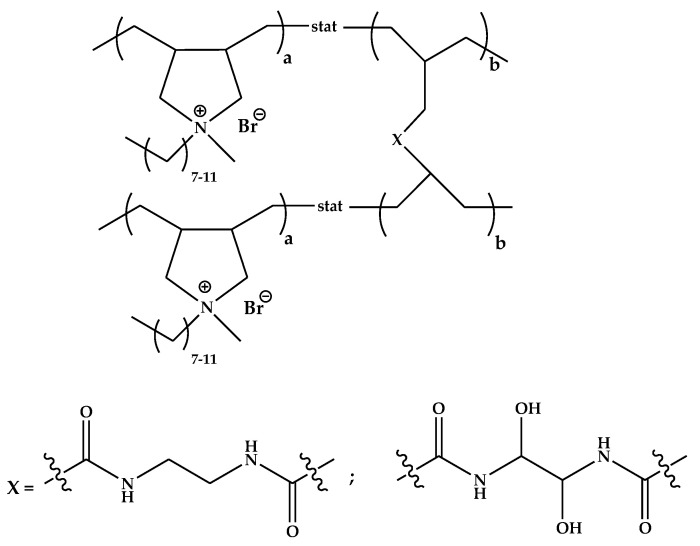
Gels based on N-alkyl-N-methyldiallylammonium bromides (adapted and redrawn from [97]).

Cationic hydrogels (Figure 10), obtained by cyclocopolymerization of hydrophobically modified dialkyldiallylammonium bromides in the presence of several multifunctional cross-linking monomers were tested on hamsters as BASs [97].

The nature of cross-linking monomers (dihydroxyethylenebisacryl amide, ethylenebisacrylamide and methylenebisacrylamide), their concentrations, and the length of the alkyl chains of the monomers were the factors that influenced polymeric hypocholesterolemic activity. The hydrogels, obtained by using flexible cross-linkers with hydrophilic functional groups in their structure (dihydroxyethylene bisacrylamide), showed the best results. Bile salt binding capacities increased with decreasing cross-linker concentration and C_10_ alkyl chain length of ammonium monomers.

**Figure 11:**
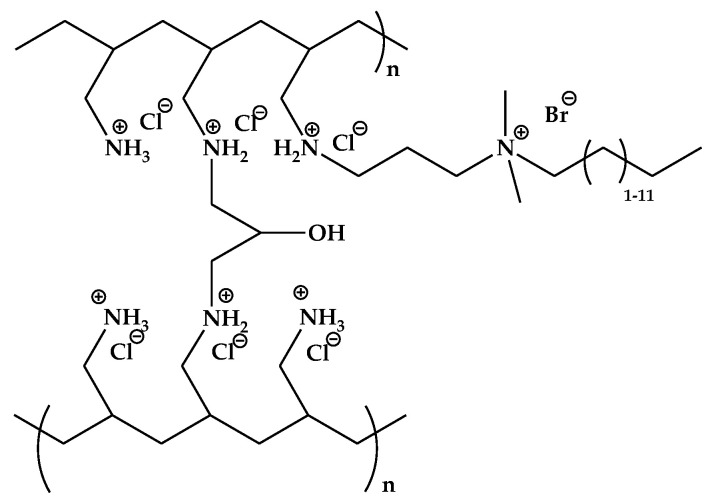
Hydrophobically modified gels based on polyallylamines (adapted and redrawn from [98]).

Several amphiphilic cationic hydrogels were prepared by the cross-linking of soluble poly(allylamine) with epichlorohydrin, followed by the alkylation of the resulted gel with different 3-chloropropyl)dimethyl-alkylammonium bromides (where alkyl was C_4_–C_12_) (Figure 11) [98]. The factors (degree of cross-linking, length of alkyl substituent) that can influence the equilibrium swelling behavior of the gels were studied using alcohols with different lipophilicity as solvents. Polymeric swelling capacity decreased as cross-linking density increased, which showed that the stiffness of cross-linked matrix governs the swelling process. Swelling ability of the polymers reached the maximum value for C_4_, decreased moderately between C_4_ and C_6_, and dropped significantly after C_8_ chain due to the collapsed structure of the gel as a result of the cooperative hydrophobic self-association of the alkyl chains. Solvent (alcohol) hydrophobicity influenced the swelling process. Thus, polymeric swelling ability was augmented with the increase in solvent lipophilicity. Amphiphilic hydrogels were assessed for their bile salt sequestration capacity by in vivo studies in a hamster animal model. The polymers’ ability to retain bile salts increased with the augmentation of chain lengths from C_8_ to C_12_ and dropped for C_14_ alkyl chain length. This behavior proved that ionic interactions together with the hydrophobic ones played significant roles in bile salts sequestration by these amphiphilic polyammonium hydrogels.

**Figure 12:**
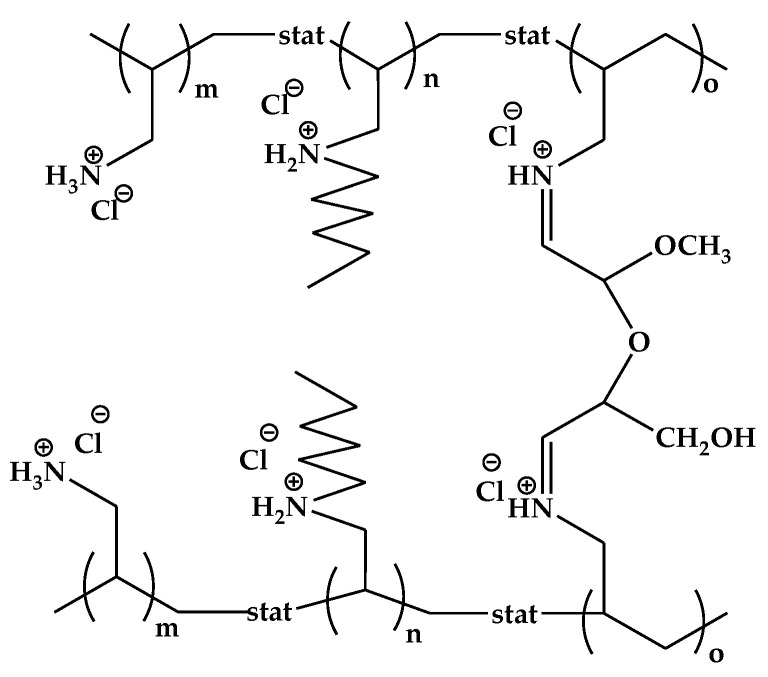
Copolymer of allylamine hydrochloride and allylhexylamine hydrochloride (adapted and redrawn from [99]).

An amphiphilic copolymer (Figure 12) synthesized by copolymerization of allylamine hydrochloride with allylhexylamine hydrochloride was cross-linked with a double aldehyde obtained by the oxidation of methyl-D-glucopyranoside by sodium periodate [99]. A molar ratio between allylamine hydrochloride and allylhexylamine hydro- chloride of 90.6/9.4 and a weight percentage of 2.75 wt% for the cross-linker allowed the obtaining of the double Schiff base cross-linked hydrogel with the optimal composition for sodium glycocholate adsorption. Isothermal titration calorimetry showed that ionic interactions and hydrophobic associations were both involved in bile salt sorption. Langmuir isotherm and pseudo-second-order kinetic model were the best fits to the experimental adsorption data, while 859.63 mg/g was the maximum adsorption capacity and only 2 h was enough for amphiphilic polymers to reach the adsorption equilibrium.

##### Molecular Imprinting Polymers (MIPs)

Molecular imprinting is based on the formation of a complex between a template and a functional monomer. In the presence of a large excess of the cross-linking agent, a 3D polymer network results. After polymerization, the template is removed from the polymer, leaving specific recognition sites complementary in shape, size and chemical functionality to the template molecule. Thus, the resultant polymer recognizes and binds selectively only the template molecules. The primary benefits of MIPs are their great selectivity and affinity for the target molecule employed in the imprinting method [100,101]. MIPs have higher physical toughness, resistance to high temperature and pressure and inactivity against acids, bases and organic solvents. Additionally, the cost of synthesis is low, and they can retain their recognition ability for several years at ambient temperature thanks to their long-lasting storage life [54]. The interactions between monomer and template can be covalent or non-covalent. If template and monomer are linked by covalent bonds, the resulting complex is stable with improved imprinting efficiency. This method is restricted to target molecules holding functional groups capable of reversible covalent bonds. If the complex is formed by physical forces (hydrogen bonds, van der Waals forces, hydrophobic interactions), the efficacy is lower. This type of interaction is required in the case of non-covalently imprinted stationary phases that afford quick sorption/desorption processes.

The hybrid imprinting method combines some of the benefits of both covalent and non-covalent approaches by using a covalent template structure in the polymerization step, but a non-covalent binding. MIPs obtained by hybrid method have a high capacity for binding and good selectivity for the guest molecule. Selectivity comes from the imprinting of templates with certain shapes and spatial arrangements of functional groups. This process is a reliable way to create MIPs that can recognize the target molecules specifically. This innovative method was reported for cholesterol-imprinted polymers using cholesteryl(4-vinyl)phenyl carbonate as template molecule [102]: (4-vinylphenyl) carbonate ester was copolymerized with ethylene glycol dimethacrylate by thermally initiated free radical polymerization using an azo-initiator (AIBN). Basic hydrolysis cleaved the carbonate bonds, and the remaining phenolic residues from the polymer cavities were able to bind cholesterol via hydrogen bonds.

Huval et al. synthesized cholic acid-imprinted polymers using poly(allylamine) cross-linked with epichlorohydrin and having sodium cholate as template molecule [103]. Two imprinted polymer networks were obtained by using two different concentrations of sodium cholate. When compared to the control polymer, MIPs synthesized with higher concentrations of cholic acid showed greater binding capacity and cooperativity for the template.

Cholic acid-imprinted polymer was synthesized by a hybrid imprinting method using the polymer-containing monomer 3α-methacryloyl cholic acid methyl ester as template and ethylene glycol dimethacrylate as cross-linker [104]. Polymeric carboxylic acid groups, strategically positioned in the cavities and acquired by removing the template after basic hydrolysis, allowed the selective binding of cholic acid via hydrogen bonds and size-specific binding. The maximal binding capacity of MIP was significantly higher than that of the non-imprinted polymer. The Scatchard method confirmed the occurrence of two dissimilar binding sites. This implied that carboxylic groups from imprinted polymeric cavities showed higher affinity and better recognition selectivity for cholic acid than for other analogous products.

In another approach to bile acid-imprinted polymers, radical polymerization of six different acrylamides with ethylenebisacrylamide as cross-linker and cholic acid acrylate as template was performed by using a molar ratio of 10:2:1 between monomer, cross-linker and template [105]. The template was separated from the polymer by ester aminolysis using an aqueous solution of N,N-dimethylethylenediamine. The obtaining tertiary amino end groups were quaternized to enable BA attachment.

**Scheme 1:**
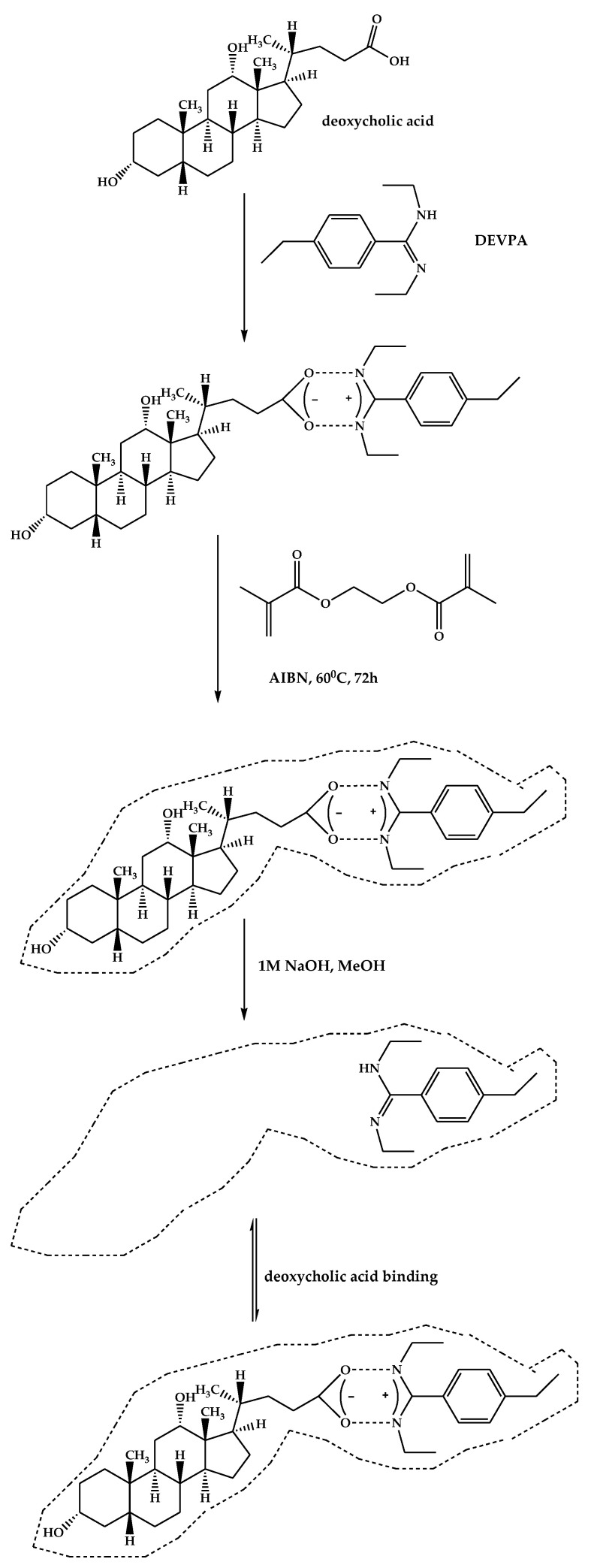
Synthetic route for deoxycholic acid–molecular imprinting polymer (adapted and redrawn from [107]).

Molecular imprinting technology and computational modeling can be coupled to create polymeric gels with a high affinity for cholic acid in aqueous media [106]. The computational modeling detected N-(3-aminopropyl)-methacrylate hydrochloride, N,N-diethylamino ethyl methacrylate and ethylene glycol methacrylate phosphate as the most suitable monomers, with a great affinity for cholic acid, from a virtual library of 18 monomers which were often used for MIP synthesis. The molar ratio between cholic acid and the functional monomer was the same (1:4), but the cross-linking degree varied by the modification of the cross-linker (ethylenebisacrylamide) content. Experiments certified that the cross-linking degree was the main parameter in projection of MIP. The best cholic acid uptake, predicted by computational modeling and proved by experimental results, was recorded by N-(3-aminopropyl)-methacrylate hydrochloride (APMA^.^HCl)-based networks, the results being comparable to those obtained by using colestipol. Anticipated by computational technique, the great results acquired by APMA^.^HCl-based gels could be explained by ionic attraction between the carboxylic group of BA and the protonated amine group of methacrylate hydrochloride and also by the hydrophobic interactions between steroid skeleton and polymeric cross-linking points which afforded BA retention. However, the difference between the results obtained by using imprinted and non-imprinted APMA^.^HCl-based gels were not substantial, which confirmed that the imprinting effect was less essential. The Freundlich model adsorption isotherm showed that the imprinting process enhanced the number of high-affinity binding sites only when the affinity of the monomer for the target molecule was not very high. The polymers’ ability to serve as sodium cholate traps was examined in phosphate buffer pH 7.4 in a dynamic mode, in an effort to mimic the environment in the gut. In comparison to the control polymers, APMA·HCl-based networks showed the highest capacity for cholic acid binding. Numerous MIPs (Scheme 1) used as BASs for the purpose of diagnosing and treating patients with diseases related to BA (atherosclerosis, liver and GIT diseases) were patented [107].

Functional monomers, used in the synthesis of imprinting gels, were: (a) 2-, 3- and 4-vinyl-2-hydroxypyridine and N,N′-diethyl(4-vinylphenyl) amidine; (b) (N,N′-diethyl(4-vinylphenyl) amidine); (c) acrylamide, methacrylamide, N-methyl(meth)acrylamide derivatives or analogues; (d) 2-, 3- and 4-vinyl-2-hydroxy pyridine, N,N’-diethyl (4-vinyl phenyl) amidine. As cross-linking agents were employed: ethyleneglycoldimeth acrylate, N,N′-diacryloyl- or N,N′-dimethacryloyl ethylenediamine, N,N′-diacryloyl- or N,N′-dimethacryloyl 1,3-diamino benzene, N,N′-diacryloyl- or N,N′-dimethacryloyl 1,4-diaminobenzene; a diacrylate or dimethacrylate of 1.2-, 1.3-, or 1,4-dihydroxy benzene, N,N′-(4-vinylbenzoyl)-1.

There are no significant data on effective BA binding in in vivo conditions by MIPs due to the complex conditions in the GIT (high concentration of low molecular ions and active reabsorption of BA by enterohepatic circulation).

##### Polysaccharide-Based BASs

Avicel 102, a microcrystalline cellulose obtained by partially depolymerized α-cellulose [108], was cross-linked by using different amounts of 1-chloro-2,3-epoxy propane, followed by amination with N, N-diethyl-2-chloroethylamine hydrochloride or 1-(N, N, N-triethylamino)-3-chloro-2-propanol [109]. Another synthetic route was the amination of cross-linked Avicel 102 with 3-(N, N-diethylamino)-1, 2-epoxypropane. These microcrystalline cellulose-based compounds were synthesized for bile salts (sodium cholate and sodium deoxycholate) adsorption. Electrostatic interactions together with hydrophobic interactions and hydrogen bonds afforded the retention of bile salts by the polymers. The Langmuir isotherm model was used to establish the characteristics of the most efficient polymeric sorbent. Increased amino group content of the resins favored the adsorption process by increasing the electrostatic interactions, but decreased the affinity of the cross-linked polymers for sorbates. Optimum polymeric water uptake (2.5–2.8 g water/g dry resin) also ensured the inclusion of BA anions in the resin network. Another factor that influenced bile salt adsorption was the sorbate hydrophobicity. Dihydroxy bile salts were retained more strongly by aminated cross-linked polymers than trihydroxy ones.

The binding capacity of sodium salts of different BAs (cholic, deoxycholate, glycocholic, and taurocholic acids) by DEAE-Granocel cellulose and DE-52 was investigated and compared with that of commercial cholestyramine [110]. DEAE-Granocel is a macroporous anion exchange resin prepared by the regeneration of cellulose from cellulose diacetate followed by amination with 2-diethylaminoethyl chloride hydrochloride [111] while DE-52 is a preswollen microgranular anion exchange resin based on diethylaminoethyl cellulose. In vitro retention of bile salts was achieved at pH ranging between 4 and 6. The adsorption equilibrium on cellulose-based sorbents was reached in approximately 30 min, while the maximum of adsorption on cholestyramine was accomplished in about 1 h. DEAE-Granocel exhibited the greatest sorption capacity—seven times that of commercial BASs. The electrostatic interactions between cellulose-based sorbents having diethylaminoethyl groups and ligands were the major driving forces in retention process.

**Figure 13:**
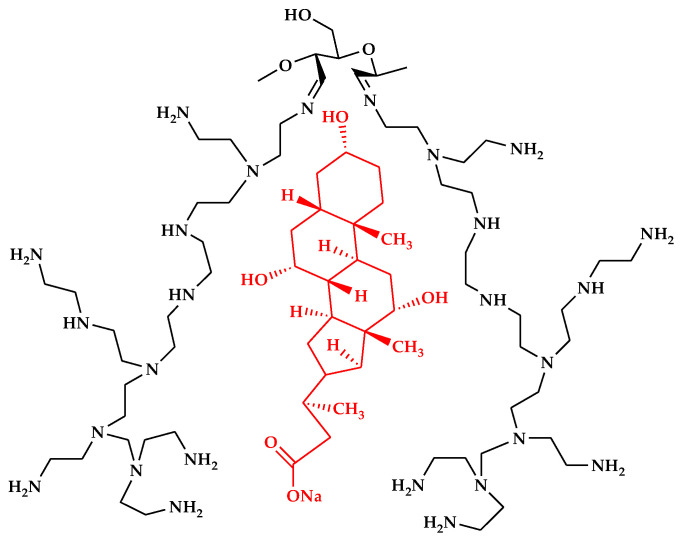
Sodium cholate sorption by polyethylenimine cellulose (adapted and redrawn from [112]).

Dialdehyde cellulose obtained from cellulose powder oxidized with NaIO_4_ was modified with hyperbranched polyethylenimine, and the resulting solid cytocompatible polymer with numerous amino groups proved to be a very good sequestrant for sodium cholate, with a maximum sorption capacity of 569.7 mg/g, which was not significantly reduced in the presence of competing ions (NaCl) (Figure 13) [112].

A detailed in vitro study investigated the influence of the chemical structure, nature of polymeric sorbent and its water uptake values for cholic acid adsorption by using aminated dextran, pullulan and microcrystalline cellulose [113].

**Figure 14:**
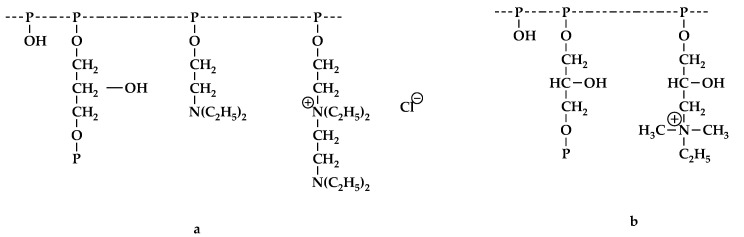
Chemical structure of cationic hydrogels based on different polysaccharides (P-OH = dextran, pullulan, cellulose) (adapted and redrawn from [113]).

In vitro dissociation of ionic complex polymer-BA was also examined. Polysaccharides, cross-linked with epichlorohydrin, were aminated with N, N-diethyl-2-chloroethylamine hydrochloride (Figure 14a) and glycidyl dimethylethyl ammonium chloride (Figure 14b) for the obtaining of tertiary amino and/or quaternary ammonium groups. According to Langmuir affinity constants, ligand–sorbent affinity depended on polymeric support and increased in the following order: cellulose < pullulan < dextran. Increased amino groups content of the resins favored the BA retention but decreased the affinity of the cross-linked polymers. Another factor that influenced the adsorption process was the swelling capacity of the sorbent, which depended on the cross-linking density. Water retention of 3–4 g/g for dextran-based polymers afforded the best fit of polymeric pore size to the dimensions of cholic acid molecules and also determined the slowest rate of dissociation of its complex with BA.

In vivo preliminary studies in which aminated polymers based on dextran and microcrystalline cellulose were orally administered to normolipemic rats showed a higher decrease in TC, atherogenic LDL, VLDL, and TG levels for dextran-based polymers compared to microcrystalline cellulose [114]. Cholestyramine was also tested. The lowest reduction in TC, LDL+ VLDL and TG levels was found for commercial BASs. Plasma levels of transaminases and α-amylases were not modified by the tested polymers, which indicated that hepatic and pancreatic functions were not affected. Intestinal transit was not modified, and no intestinal occlusions were observed.

**Figure 15:**
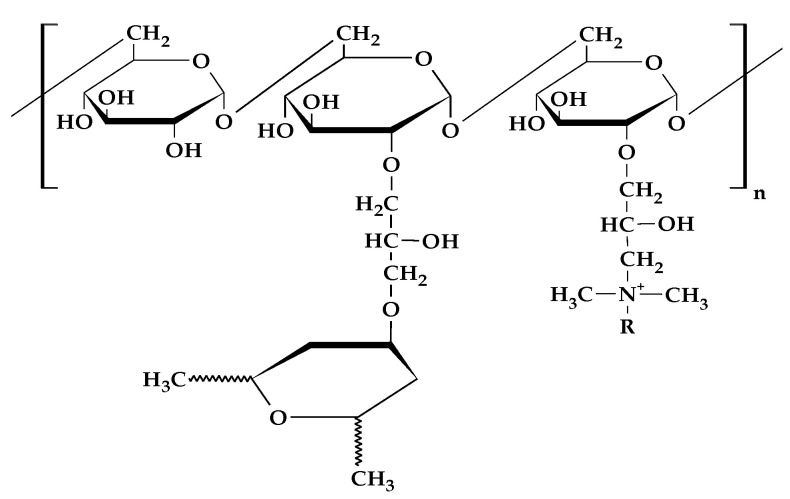
Chemical structure of cationic amphiphilic gels based on dextran (R = ethyl, butyl, octyl, dodecyl, DS = 100x/(x + y)) (adapted and redrawn from [115,116]).

Cationic amphiphilic dextran-based polymers (Figure 15) [115,116] synthesized by amination of cross-linked polysaccharide with an equimolar mixture of epichlorohydrin and dimethylalkylamine (alkyl = ethyl, butyl, octyl, dodecyl) and with 20–25 mol% ammonium chloride side chains were used for the adsorption of sodium salts of different BAs (cholic, glycocholic and deoxycholic) in water and 10 mm NaCl solutions [117]. A nearest-neighbor-interaction model, which allowed the analysis of binding experimental data, was used for the study of the factors that can influence the binding process (length of the alkyl substituent of the gel, ionic strength, BA lipophilicity). The augmentation of the length of the alkyl substituent increased the binding process, but decreased the cooperativity of bile salt retention. In contrast, ionic strength decreased bile salt adsorption, but increased binding cooperativity. For short alkyl substituents (ethyl, butyl), the interactions between ligand and adsorbent were mainly electrostatic and aggregation occurred mostly via single-component micelles of bile salt, while for long substituents (octyl, dodecyl), interactions were mostly lipophilic and aggregation happened mainly through mixed micelles formed between side chains of the polymer and bile salts. The binding constants were higher for the adsorption of dihydroxylic bile salts than trihydroxylic ones due to the stronger hydrophobic interactions between hydrogel and ligand. Studies concerning sorption of sodium cholate in the presence of electrolytes (NaCl, NaHCO_3_ or KH_2_PO_4_) showed that the selectivity towards bile salt depended on the nature of alkyl substituent and increased with the augmentation of its length [118].

In vitro sorption of sodium salts of four BAs (glycocholic, cholic, taurocholic, and deoxycholic) by dextran-based hydrogels bearing quaternary ammonium pendant groups was evaluated as a function of alkyl substituent length and bile salt lipophilicity [119]. Adsorption tests were conducted in phosphate-buffer solutions (pH 7.4) containing one bile salt (individual sorption) or mixtures of several bile salts (competitive sorption). Individual sorption studies showed the same behavior previously found in other adsorption studies of bile salts by dextran-based gels with N-alkyl-N,N-dimethyl ammonium chloride pendant groups: that of augmentation of the binding constants with the increase in the length of alkyl substituent while the binding cooperativity decreased. The competitive sorption studies revealed good affinity of the hydrogels for both dihydroxylic and trihydroxylic bile salts. The binding constants for the retention of bile salts by gels with ethyl and dodecyl as alkyl substituent were 20 and 30 times higher, respectively, than those obtained for commercial cholestyramine under the same adsorption conditions.

**Figure 16:**
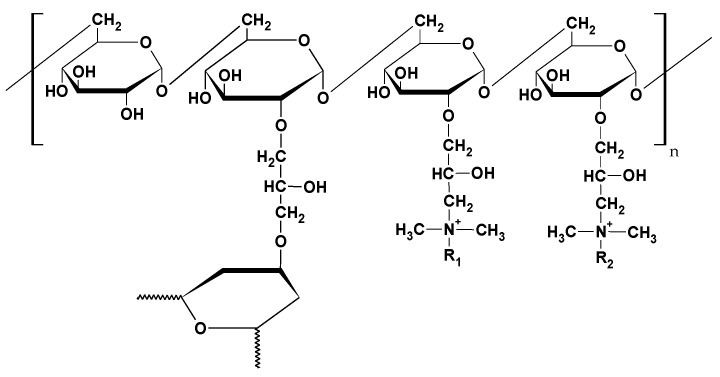
Chemical structure of dextran gels (R_1_ = dodecyl/hexadecyl; R_2_ = ethyl) (adapted and redrawn from [120,121,122].

Dextran hydrogels, with two types of pendant trialkyl quaternary ammonium chloride groups with different hydrophobicity endowed by the length of one alkyl substituent (ethyl and dodecyl/hexadecyl, respectively) (Figure 16) [120,121,122], were designed to have a similar total content in ammonium groups, but different content in hydrophobic groups. They were evaluated as adsorbents for sodium cholate and sodium deoxycholate in water and 10 mM NaCl solutions [123,124].

Adsorption mechanisms and characteristics of the most efficient polymeric sorbent were clarified by using various isotherm models (nearest-neighbor interaction, Langmuir, Freundlich, Dubinin–Raduskevich, Sips and Hill). The hydrogel–ligand affinity increased with the augmentation of side-chain hydrophobic group percentage and polymeric water uptake, as well as with the increase in bile salt lipophilicity. Ionic strength decreased bile salt retention, particularly for the hydrophilic hydrogels. The polymer with 25 mol% pendant dodecyl group adsorbed the maximum amount of bile salts (1051 mg NaCA/g and 1138 mg NaDCA/g) due to its optimal hydrophilic-lipophilic balance, high charge density and water retention values. All amphiphilic gels exhibited better affinity and strength of binding than cholestyramine, the polymer with the best adsorption results having five times the affinity and 247 times the binding strength of commercial BASs.

A similar approach for dual polysaccharide modification was applied by Zhu et al. [125] for the synthesis of BASs based on microfibrillated cellulose. In vitro adsorption studies of BA highlighted the same dependence of performances in hydrophobic groups, but the maximum adsorption was modest (50% of that of cholestyramine), perhaps due to low content in the total amino groups (about 7 mol%).

Quaternized methylans were tested in vitro as adsorbents for various sodium salts of different BAs (glycocholic, glycodeoxycholic, glycochenodeoxycholic, taurocholic, taurodeoxycholic and taurochenodeoxycholic acids) [126]. Adsorption studies were carried out in 15 mM aqueous NaCl solutions (pH 5.4–5.6) containing one bile salt (individual sorption) or mixtures of several bile salts (competitive sorption). The methylan-based adsorbent was obtained by two different synthetic pathways. The first one consisted in the amination of the polysaccharide with several dialkylaminoalkylchlorides (diethylaminoethylchloride, dimethylaminoethylchloride and dimethylaminoisopropyl chloride) followed by quaternization with methyl iodide.

**Scheme 2:**
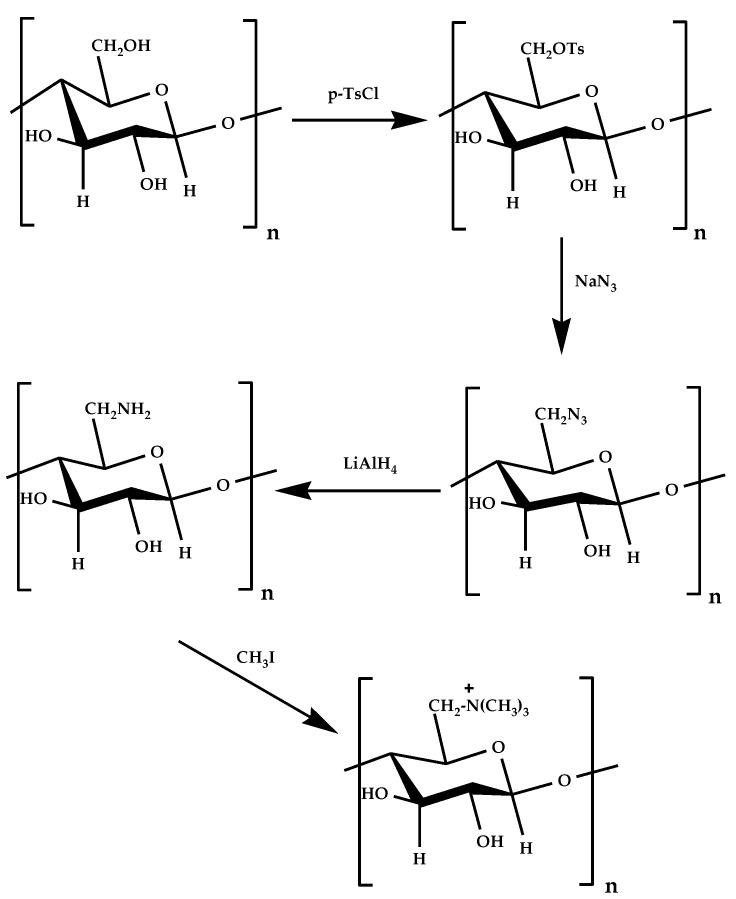
Amination of methylan followed by quaternization (adapted and redrawn from [126]).

The second synthetic route was composed of tosylation of methylan, followed by azidation and then reduction with LiAlH_4_, which afforded methylan-NH_2_, followed by its quaternization with methyl iodide (Scheme 2). The polymers showed binding capacities higher than those of cholestyramine. The retention of bile salts increased with the augmentation of polymeric alkyl length chain, proving that the hydrophobic interactions together with ionic ones were the forces that afforded bile salt sequestration.

The ability of cyclodextrins to selectively bind hydrophobic compounds in their hydrophobic cavities was also exploited for the design of new and efficient BASs. Copolymers obtained by the cross-linking of β-cyclodextrin with different amounts of epichlorohydrin using a suspension polymerization technique were examined for their capacity to bind sodium salts of BA (cholic, glycocholic, taurocholic and chenodeoxycholic acid), individually and competitively, in phosphate-buffered solutions [127].

**Figure 17:**
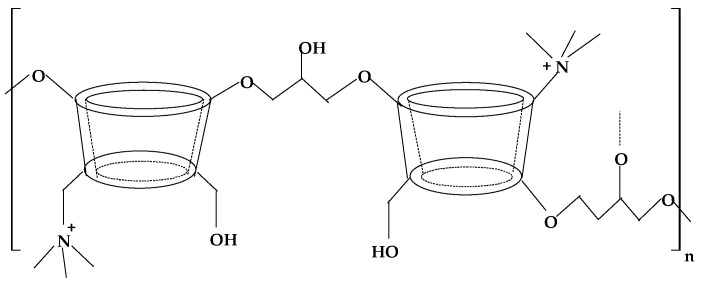
Structure of quaternized poly(cyclodextrin) resin (adapted and redrawn from [127]).

In the case of polymeric cyclodextrins, the host cavity size compared with that of the guest molecules is of notable importance, the hydrophobic interactions between adsorbent and ligand playing a secondary role in the adsorption process. A lower degree of cross-linking will improve resin binding ability, as a less dense matrix will favor the formation of the inclusion complexes between β-CD cavities and BA anions. The binding of a smaller bile salt anion (chenodeoxycholate) by the polycyclodextrin was much more efficient than that of a bulkier (cholate) one, competitive binding showing that 60–70% of β-cyclodextrin cavities were filled by chenodeoxycholate and only 10% by cholate. The higher percentage of chenodeoxycholate retention in cyclodextrin cavities could also be explained by its higher lipophilicity compared to that of cholate. Poly(β-cyclodextrin) resins and their quaternized polymers (Figure 17) were tested for their ability to bind sodium salts of different BAs (cholic, glycocholic and chenodeoxycholic), individually and competitively, in phosphate-buffered solutions [128]. The polymers were synthesized by the cross-linking of β-cyclodextrin with different amounts of epichlorohydrin, succeeded by tosylation and amination with trimethylamine.

Chenodeoxycholate adsorption was more efficient than that of cholate, the explication being the smaller size and increased hydrophobicity of chenodeoxycholate anion [127]. The retention capacity of poly(β-cyclodextrin) and quaternized poly(β-cyclo-dextrin) decreased with the augmentation of temperature, and this factor had a greater effect for quaternized poly(β-cyclodextrin), proving that temperature influenced the electrostatic interactions during bile salts adsorption. On the contrary, hydrophobic interactions were not influenced as much by temperature.

Divinyl sulfone was chosen as cross-linking agent for β-cyclodextrin, starch and dextrin homopolymers, as well as equivalent β-cyclodextrin/starch and β-cyclodextrin/dextrin heteropolymers [129]. The resulting library of 22 homo- and heteropolymers was tested against cholesterol, cholic and deoxycholic acid to evaluate polymeric ability to adsorb BA [130]. As was found in previous works, which studied bile salt sequestration by poly(cyclodextrin) resins [127,128], good complexation was obtained by polymers with a low degree of cross-linking. Among the homopolymers with the same degree of cross-linking, the best bile salt retention results were achieved by using β-cyclodextrin-based ones. The heteropolymers of starch and dextrin with β-cyclodextrin displayed higher BA adsorption capacities than β-cyclodextrin homopolymer. The adsorption capacity of cholesterol for two low cross-linked β-cyclodextrin–starch and β-cyclodextrin–dextrin heteropolymers was tested on hypercholesterolemic male Wistar rats. Both polymers had comparable abilities to retain cholesterol (1.61–1.75 mmol/L). Furthermore, TG values were decreased from 1.25 mmol/L to 0.74–0.89 mmol/L. Transaminases and creatinine levels were not modified by the ingestion of heteropolymers, which suggested an absence of toxicity for these resins.

## 3. Summary and Future Perspectives

Untreated hyperlipidemia, the lipid disorder characterized by an elevated level of low-density lipoprotein, produces atherosclerosis in the arteries, which leads to heart attack and stroke. Atherosclerosis, the principal cause of cardiovascular disease, is the leading cause of death worldwide. Commercial BASs have reduced effect due to their low binding selectivity for trihydroxy BA and lower stability of the BA–BAS complex, which can dissociate in its passage through the ileum and colon. Commercial BASs also produce side effects, such as queasiness, costiveness, and flatulence, due to the high doses necessary to suppress dissociation and to obtain a healing effect. Consequently, there is an acute demand for new BASs with greater selectivity for BA over other anions, enhanced binding ability and stability to dissociation in the GIT. This review describes various synthetic routes used in obtaining new effective natural and synthetic gel-based BASs that affords comprehension of the relationships between their chemical structure and ability to bind BA. There are three types of polymeric BASs. The first type consists of amphiphilic polymers with quaternary ammonium groups or polymers containing secondary and tertiary amino groups, which have all amino groups ionized at the pH of the small intestine. These BASs are based either on biopolymers, such as cellulose, dextran, pullulan, and methylan, or synthetic polymers, such as poly((meth)acrylates/acrylamides), poly(alkylamines), poly(allylamines), and vinyl benzyl amino polymers. In vitro studies showed various key factors that help in the augmentation of BAS efficiency: (1) cationic charge density for the purpose of preserving electrostatic interactions with BA; (2) the occurrence of hydrophobic pendant groups with the aim of assuring lipophilic interactions with steroid nuclei of BA; (3) flexibility of the polymeric skeleton, which favors the adsorption process; (4) the cross-linking degree, which assures suitable swelling properties for gel-based adsorbents; and (5) the presence of H-bond-forming groups that strengthen BA sorption. Two copolymers (SK&F 97426-A and DMP 504) were tested on hamsters or hyperlipidemic patients in in vivo studies, showing a reduction several-fold higher for cholesterol and different lipoprotein levels compared to that of cholestyramine. The second type of BASs were polymeric cyclodextrins, which were used as non-specific sequestrants due to their unique capacity to form quite strong inclusion complexes with BA. The deep penetration of BA into the cavity of cyclodextrins, which ensures a high stability of the constituting complex, can be explained by the size of β-cyclodextrin cavities, which is similar to the corresponding part of BA, and by the specific structural characteristics of BA (the occurrence of hydroxyl groups at C^3^, C^7^ and/or C^12^ on the hydrophobic steroid backbone). In vitro and in vivo studies showed the high potential of polymeric cyclodextrins as BASs due to their strong affinity to BA and great reduction of different forms of lipids. The third type of BASs are MIPs. Despite the fact that they showed higher affinity in vitro to BA due to their adapted binding sites, their binding strength was lower than other BASs. Furthermore, there are no substantial data on efficacious BA binding by MIPs in in vivo conditions. Because an ideal BAS is still missing in the market, studies for finding new biomaterials with high binding capacity and affinity in BA retention as well as a safety profile must be continued. Future studies should be oriented towards the use of polymerization techniques that permit the obtaining of custom-made materials, thus making the growth of a new BAS generation possible.

## Data Availability

Not applicable.
